# Adaptive mutations promote hepatitis C virus assembly by accelerating core translocation to the endoplasmic reticulum

**DOI:** 10.1074/jbc.RA120.016010

**Published:** 2020-11-23

**Authors:** Fuxiang Zheng, Ni Li, Yi Xu, Yuanping Zhou, Yi-Ping Li

**Affiliations:** 1 Institute of Human Virology, Zhongshan School of Medicine, and Key Laboratory of Tropical Disease Control of Ministry of Education, Sun Yat-sen University, Guangzhou, China; 2 Department of Pediatric, Guangzhou Women and Children’s Medical Center, Guangzhou Medical University, Guangzhou, China; 3 Department of Infectious Diseases, Nanfang Hospital, Southern Medical University, Guangzhou, China; 4 Department of Infectious Disease, The Fifth Affiliated Hospital of Sun Yat-sen University, Zhuhai, China

**Keywords:** hepatitis C virus envelopment, morphogenesis, protein–protein interaction, lipid droplets, endoplasmic reticulum, ER, endoplasmic reticulum, FFU, focus forming unit, HCV, hepatitis C virus, HRP, horseradish peroxidase, LD, lipid droplet, TM, transmembrane domain

## Abstract

The envelopment of hepatitis C virus (HCV) is believed to occur primarily in the endoplasmic reticulum (ER)-associated membrane, and the translocation of viral Core protein from lipid droplets (LDs) to the ER is essential for the envelopment of viral particles. However, the factors involved are not completely understood. Herein, we identified eight adaptive mutations that enhanced virus spread and infectivity of genotype 1a clone TNcc in hepatoma Huh7 cells through long-term culture adaptation and reverse genetic study. Of eight mutations, I853V in NS2 and C2865F in NS5B were found to be minimal mutation sets that enabled an increase in virus production without apparently affecting RNA replication, thus suggesting its roles in the post-replication stage of the HCV life cycle. Using a protease K protection and confocal microscopy analysis, we demonstrated that C2865F and the combination of I853V/C2865F enhanced virus envelopment by facilitating Core translocation from the LDs to the ER. Buoyant density analysis revealed that I853V/C2865F contributed to the release of virion with a density of ∼1.10 g/ml. Moreover, we demonstrated that NS5B directly interacted with NS2 at the protease domain and that mutations I853V, C2865F, and I853V/C2865F enhanced the interaction. In addition, C2865F also enhanced the interaction between NS5B and Core. In conclusion, this study demonstrated that adaptive mutations in NS2 and NS5B promoted HCV envelopment by accelerating Core translocation from the LDs to the ER and reinforced the interaction between NS2 and NS5B. The findings facilitate our understanding of the assembly of HCV morphogenesis.

Hepatitis C virus (HCV) infection is a major cause of chronic liver diseases, including cirrhosis and hepatocellular carcinoma. Worldwide, approximately 71 million people are chronically infected with HCV, leading to ~400,000 deaths each year (1). Direct-acting antiviral agents have greatly improved the cure rate of patients with hepatitis C; however new challenges exist. To date, no HCV vaccine is available. The large number of undiagnosed patients and reinfections after cure and the limited accessibility and the unaffordability of direct-acting antiviral agent treatment in many underdeveloped countries and regions are delaying the global elimination of hepatitis C. Data from the World Health Organization show that annually 1.7 million people were infected by HCV from 2014 to 2018. Thus, HCV infection continues to be a health threat to the world population.

The HCV genome is a single-stranded, positive-sense RNA of approximately 9.6 kb in length, consisting of one open reading frame and 5′ and 3′ untranslated regions (UTRs). The open reading frame is translated into a single polyprotein, which is co- and post-translationally processed to generate three structural proteins, Core, E1, and E2, and seven nonstructural proteins, p7, NS2, NS3, NS4A, NS4B, NS5A, and NS5B. Structural proteins make up the basal structure of the virion, and nonstructural proteins are primarily responsible for RNA replication and virus assembly (2, 3).

Although great progress has been achieved in the study of the HCV life cycle after the development of HCV replicons (4), pseudotyped particles (HCVpp) (5), and infectious HCV cell culture systems (HCVcc) (6, 7, 8, 9), the late stages, including assembly, maturation, and release are not completely understood. Lipid droplets (LDs) and the endoplasmic reticulum (ER) are the sites for HCV envelopment to generate infectious mature virus particles (10, 11). Orchestrated by viral and host proteins, mature Core proteins oligomerize in the ER and are translocated to the LDs, where viral RNAs are encapsidated to form nucleocapsids. The nucleocapsids are then budded to the ER membrane to acquire phospholipids incorporated with E1/E2 heterodimers (12, 13). The retrieval of Core back onto the ER is a key step for efficiently producing infectious virus particles (11, 14). However, the factors involved in Core translocation between the LDs and the ER are elusive. The virus particles are further enveloped by apolipoproteins and very-low-density lipoprotein to gain infectivity and specific density (15, 16), and the particles with a buoyant density of ∼1.10 to 1.12 g/ml display the highest infectivity (6, 7, 17).

Interactions between nonstructural proteins play important roles in infectious viral particle assembly. NS2 plays a critical role in bridging the assembly of the nucleocapsids by coordinating with p7, NS3, NS5A, and E1/E2 complexes (18, 19, 20, 21, 22, 23, 24). The N-terminal transmembrane domain (TM) of NS2 inserts into the ER membrane, whereas the C-terminal region resides in the cytoplasm and is involved in the activity of zinc-stimulated NS2/NS3 autoprotease (18, 25, 26). The protease domain of NS2, together with the N-terminal residues of the NS3 cofactor, cleaves the junction of NS2 and NS3 (26). The NS5B is an RNA-dependent RNA polymerase primarily responsible for viral RNA replication. Several adaptive mutations in NS5B increase viral infectivity titers without apparently affecting RNA replication (9, 27, 28) or polymerase activity (9), indicating that NS5B also plays a role in HCV morphogenesis. However, the molecular mechanisms involved have not been reported. A genetic synergistic effect between NS2 and NS5B has been indicated by mutations that appeared in these two proteins (9, 27). *In vitro* glutathione *S*-transferase pull-down and coimmunoprecipitation (Co-IP) assays have shown the interaction of NS2 and NS5B (29); however, the binding domains and functional role of this protein–protein interaction have not been explored.

The development of HCVcc of different genotypes allows us to dissect the HCV life cycle, in which adaptive mutations could provide the clues. Taking advantage of culture adaptive mutations, a number of studies have uncovered the important details of the HCV life cycle, including RNA replication (23, 27, 30, 31, 32, 33, 34), viral assembly (7, 9, 23, 31, 32, 33, 34, 35, 36, 37), and particle maturation and release (28, 38, 39). However, most of the studies were based on genotype 2a JFH1 clone or JFH1-based HCV chimeras (6, 7, 8). Viral genotype is considered as one of the factors affecting the outcome of hepatitis C (40). Genotype 1 is the most prevalent and accounts for more than 50% of global HCV infections (41, 42, 43, 44). Knowledge from the study of the genotype 1 virus would be most beneficial to the understanding of HCV pathogenesis. In this study, by using the infectious genotype 1a clone TNcc that we developed previously (9), we identified adaptive mutations in NS2 and NS5B and demonstrated that both mutations cooperatively promoted the envelopment of HCV assembly. Mechanistic studies further revealed that these two mutations accelerated Core translocation from the LDs to the ER and reinforced the interaction between NS2 and NS5B, as well as Core and NS5B. This study provides new evidence toward elucidating the late steps of HCV morphogenesis.

## Results

### Enhanced infectivity of HCVcc in Huh7 cells was concomitant with the emergence of mutations

**Figure 1:**
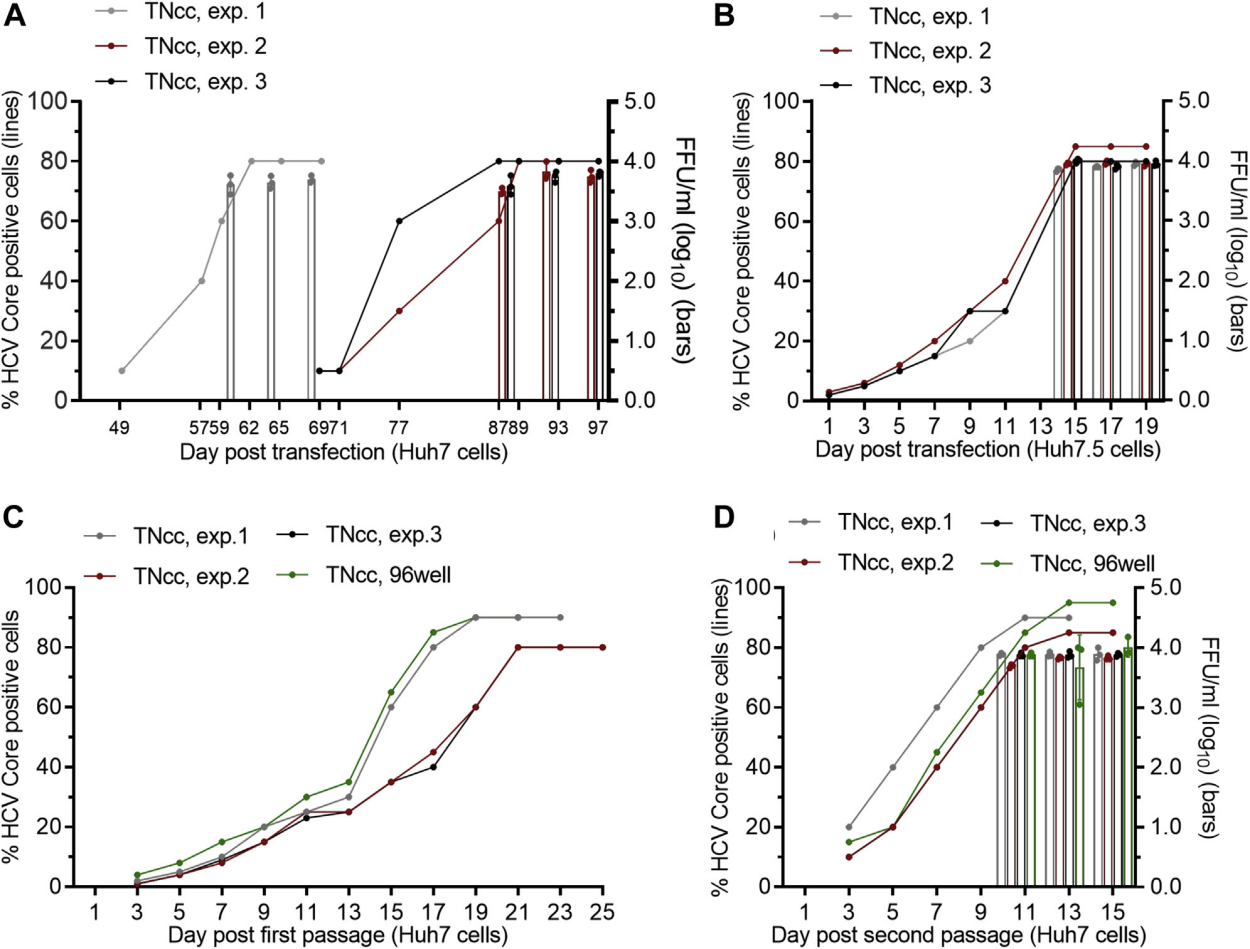
**TNcc was attenuated in Huh7 cells, but the infectivity was enhanced by viral passage.***A* and *B*, equal amounts of RNA transcripts (5 μg) of TNcc were transfected into Huh7 cells (*A*) and Huh7.5 cells (*B*). The percentage of HCV Core-positive cells was monitored (*left y-axis*, *lines*) by immunostaining, and infectivity titers of culture supernatant were determined by FFU assay (*right y-axis, bars*) at the indicated time points. In Huh7 cells, the virus spread was highly delayed (<10% within 49 days). *C* and *D*, the supernatants from transfected Huh7 cell cultures were passaged to naive Huh7 cells to obtain first-passage virus (passage 1), and the supernatants from the peak infection of first passage were used to infect naive Huh7 cells to obtain second-passage virus (passage 2). The infectivity titers of second-passage viruses were determined (*D*). A portion of the cells from *A*, exp.1, were diluted and seeded into a 96-well plate, and the cells from the well that showed the highest infection was expanded in the dishes grown naive Huh7 cells. Three independent experiments were performed (exp. 1, 2, and 3) for TNcc. Data are from three independent experiments and are shown as scatter ± SD.

Given the high permissiveness of Huh7.5 and Huh7.5.1 cells for HCV replication (6, 45), most of HCV replicons and infectious culture systems of different genotypes have been developed based on these two cell lines (6, 7, 9, 30, 37, 46, 47, 48, 49, 50). Genotype 1a clone TNcc is an infectious full-length clone that we developed to efficiently replicate in Huh7.5 cells (9). In an effort to further study the mechanism of TNcc replication, we attempted to adapt the TNcc to Huh7 cells. Huh7 cells are the origin of both Huh7.5 and Huh7.5.1 cells and possess wildtype RIG-I–mediated intracellular antiviral defense (6, 45, 51, 52). We produced TNcc RNA transcripts by *in vitro* transcription (5 μg) and transfected them into Huh7.5 and Huh7 cells in parallel in three independent experiments (Fig. 1). After RNA transfection, the percentage of Core-positive cells was determined by immunostaining using anti-HCV Core antibody, and viral infectivity titers were determined by focus forming unit (FFU) assay as previously described (9). The results showed that the spread ability of the TNcc was severely delayed in Huh7 cells compared with that in Huh7.5 cells, reaching peak infection (≥80% cell infected) at 62 and 14 days, respectively; the infectivity titer of Huh7-released TNcc virus was also lower (10^3.2^ FFU/ml) than that of Huh7.5 cell-released virus (10^4.0^ FFU/ml) (Fig. 1, *A* and *B*). The delay of virus spread in the Huh7 cells were also observed for the full-length genotype 2b clone J8cc-HT (47) (Supporting information Fig. S1, *A* and *B*) and intragenotypic recombinant 2a J6^5′UTR-NS2^/JFH1 (53) (Supporting information Fig. S1, *C* and *D*). These results indicate that Huh7.5-adapted HCVcc was attenuated in Huh7 cells and confirm that Huh7 cells are inefficient in supporting HCV replication (6, 45, 51).

To increase the virus infectivity, we collected culture supernatant from the peak infection and continually passaged it to naive Huh7 cells (Fig. 1, *C* and *D*). To facilitate the isolation of a highly infectious virus clone, we also passaged the virus in a 96-well plates and then selected the well with the fastest virus spread for further expansion in the dish (named “TNcc, 96well”) (Fig. 1, *C* and *D*). After the second passage, the virus spread ability and infectivity titers of TNcc in Huh7 cells (∼10^4^ FFU/ml) were increased and comparable with those in Huh7.5 cells (Fig. 1, *B* and *D*), indicating that TNcc may have acquired adaptive mutations for efficient infection in Huh7 cells. Thus, we extracted HCV RNA from passage-recovered viruses, amplified it by RT-PCR, and sequenced the PCR products. A number of mutations were identified in the Huh7-adapted TNcc from four independent experiments (Table 1), whereas no mutation was identified in Huh7.5-released virus. C2865F in NS5B was found in all four viruses, and seven mutations, including I853V (NS2), I1561T and G1328A (NS3), V1750D and I1769V (NS4B), G2239D and D2413N (NS5A), were identified in two or more viruses (Table. 1).

**Table 1:** Sequence analysis of TNcc virus after recovery from Huh7 cells

HCV	FFU/ml, log_10_		Passage (day)	NS2	NS3	NS3	NS4B	NS4B	NS5A	NS5A	NS5B
	Peak	Sequenced									
Nucleotide position											
TNcc (JX993348)^a^				2898	4324	5022	5590	5646	7057	7578	8935
TNcc nucleotide				A	G	G	T	A	G	G	G
TNcc virus^b^											
exp. 1	3.85	3.70	Passage 2 (7)	.	.	.	A	.	A	A	T
exp. 2	3.89	3.89	Passage 2 (7)	G	.	A	.	G	A	.	T
exp. 3	3.83	3.69	Passage 2 (7)	G	C	C	.	G	.	.	T
96well	4.10	3.88	Passage 3 (9)	.	C	.	A	.	A	A	T
TNcc with additional mutations^c^											
I853V	2.35	ND	Transfection (15)	G	.	.	.	.	.	.	.
G1328A	bld	ND	Transfection (15)	.	C	.	.	.	.	.	.
A1561T	bld	ND	Transfection (15)	.	.	A	.	.	.	.	.
V1750D	bld	ND	Transfection (15)	.	.	.	A	.	.	.	.
I1769V	bld	ND	Transfection (15)	.	.	.	.	G	.	.	.
G2239D	bld	ND	Transfection (15)	.	.	.	.	.	A	.	.
D2413N	bld	ND	Transfection (15)	.	.	.	.	.	.	A	.
C2865F	2.83	ND	Transfection (13)	.	.	.	.	.	.	.	T
VF	3.80	ND	Transfection (13)	G	.	.	.	.	.	.	T
ATF	3.70	ND	Transfection (27)	.	C	C	.	.	.	.	T
DVF	3.70	ND	Transfection (27)	.	.	.	A	G	.	.	T
VATF	3.60	ND	Transfection (19)	G	C	C	.	.	.	.	T
VATDNF	3.70	ND	Transfection (13)	G	C	C	.	.	A	A	T
VATDDNF	3.80	ND	Transfection (13)	G	C	C	A	.	A	A	T
VATDVDNF (8m)	3.85	ND	Transfection (13)	G	C	C	A	G	A	A	T
Amino acid position											
TNcc				853	1328	1561	1750	1769	2239	2413	2865
Change				I-V	G-A	I-T	V-D	I-V	G-D	D-N	C-F

HCV RNA was transfected into Huh7 cells, the transfected cultures were subcultured every 2 to 3 days, and culture supernatants were collected to determine the infectivity titer and sequence when the virus spread to ≥80% cultured cells (designated as transfection virus). For virus passage, the supernatant collected from peak infection of transfected culture was used to infect naive Huh7 cells and the supernatant was collected for analysis when the virus spread to peak infection (designated as passage virus). Mutations identified in sequencing reads are indicated. “.” (dot) indicates identical to the TNcc sequence.

bld, below the limit of detection; ND, not determined.

a The nucleotide and amino acid positions of TNcc shown in the table are identical to the genotype 1a strain H77 (AF009606).

b Mutations identified in the genome of passage-recovered viruses are indicated in the table.

c Mutations engineered into the TNcc mutant genomes are shaded; VF, I853V/C2865F; ATF, G1328A/I1651T/C2865F; DVF, V1750D/I1769V/C2865F; DNF, G2239D/D2413N/C2865F; VATF, I853V/G1328A/I1561T/C2865F; VATDNF, I853V/G1328A/I1561T/G2239D/D2413N/C2865F; VATDDNF, I853V/G1328A/I1561T/G2239D/V1750D/D2413N/C2865F; VATDVDNF (8m), I853V/G1328A/I1561T/G2239D/V1750D/I1769V/D2413N/C2865F.

### Mutations in NS2 and NS5B increased virus production by enhancing virus envelopment

Huh7 cells possess an intact RIG-I pathway that mediates interferon (IFN) responses (51). To explore whether Huh7-adapted TNcc viruses were more resistant to the IFN response, we infected Huh7.5 cells with Huh7-adapted viruses and treated the culture with different concentrations of IFN; in parallel, Huh7.5-derived viruses were included for comparison (Supporting information Fig. S2). The results showed that Huh7-adapted viruses from three experiments were similar to the original Huh7.5-derived TNcc in response to IFN treatment (Supporting information Fig. S2). These results suggest that the increased virus infectivity of TNcc in Huh7 cells may be attributed to an efficient viral life cycle but not viral resistance to the IFN response.

**Figure 2:**
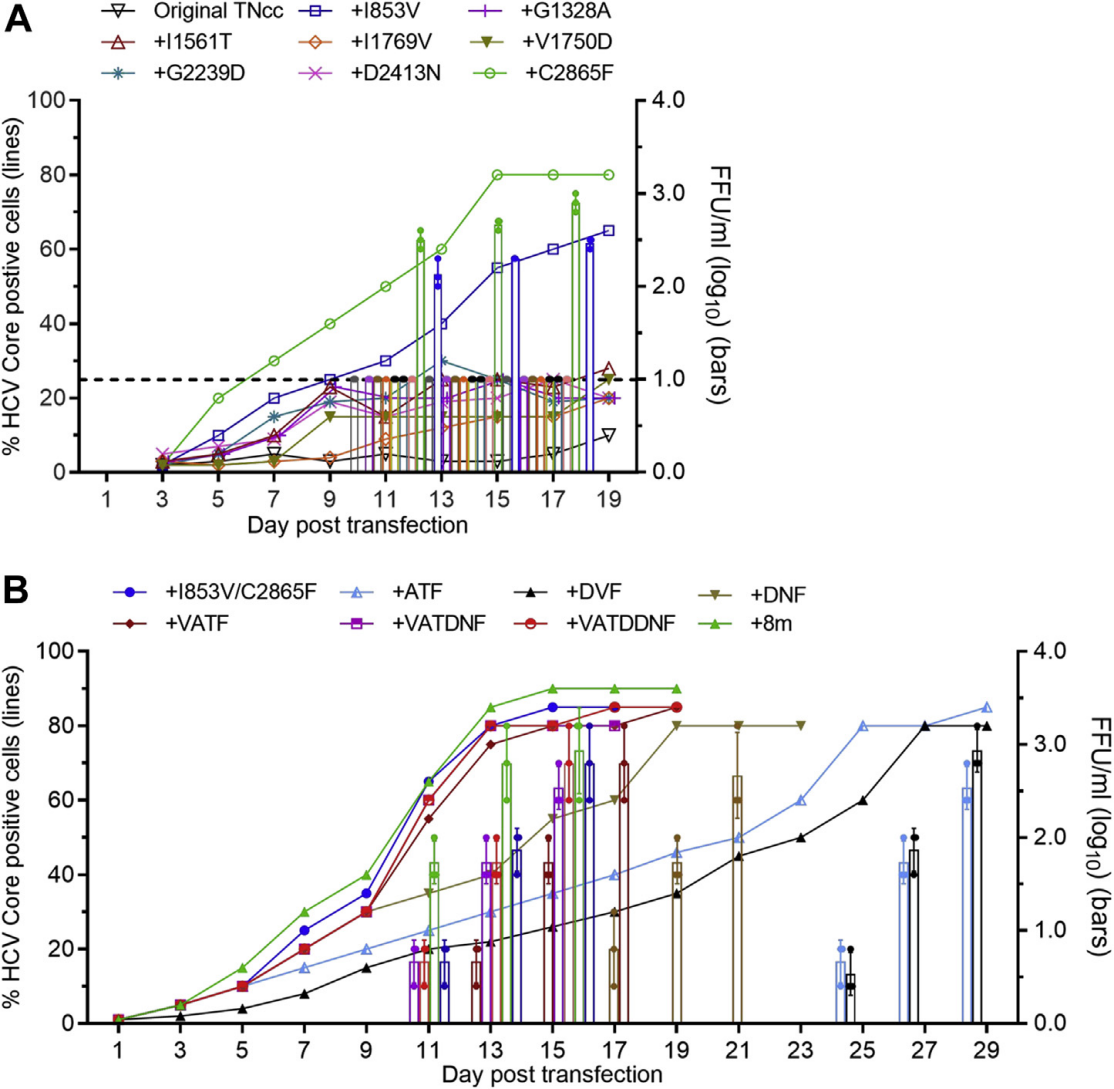
**Effects of Huh7-derived adaptive mutations on the virus spread and infectivity of TNcc virus.***A*, RNA transcripts of TNcc engineered with single (*A*) or combinations (*B*) of mutations were transfected into Huh7 cells using the original TNcc as a control. The percentage of HCV Core-positive cells and the infectivity titers of supernatant are shown at the indicated time points. Data are from three independent experiments and are shown as scatter ± SD. The *dotted line* in *A* shows the limit of detection of FFU assay. For viruses with a low infection rate, FFU was not determined. Mutations in *B* are the following: ATF, G1328A/I1651T/C2865F; DVF, V1750D/I1769V/C2865F; DNF, G2239D/D2413N/C2865F; VATF, I853V/G1328A/I1561T/C2865F; VATDNF, I853V/G1328A/I1561T/G2239D/D2413N/C2865F; VATDDNF, I853V/G1328A/I1561T/G2239D/V1750D/D2413N/C2865F; All eight mutations (8m), I853V/G1328A/I1561T/G2239D/V1750D/I1769V/D2413N/C2865F.

Next, we studied the role of adaptive mutations in the context of the complete HCV life cycle by a reverse genetic approach. We introduced eight mutations (8m), singly or in combinations, back to the TNcc and transfected the resulting transcripts into Huh7 cells, in comparison with the transfection of the original TNcc RNA (Table 1 and Fig. 2). For those TNcc carrying single mutations, mutants with I853V (NS2) or C2865F (NS5B) were more efficient than other mutant viruses, reaching peak infection at day 13 and day 15, respectively (Fig. 2, *A*). Overall, other mutations showed slight enhancement in viral spread, but the infectivity titers were below the limit of detection (Fig. 2, *A*). The combination of the I853V/C2865F was comparable with those mutants carrying four, six, seven, or all of eight mutations, reaching peak infection at day 11 (∼10^3.5^ FFU/ml), whereas other mutants with three mutations were apparently delayed (Fig. 2, *B* and Table 1). After passage to naive Huh7 cells more than 20 times, the engineered mutations were maintained in the I853V/C2865F mutant and none of the other six mutations were identified, thus indicating that the I853V/C2865F was the minimal mutation sets for the increased virus production. These data suggest that I853V and C2865F were mainly responsible for the enhanced infectivity of TNcc in Huh7 cells.

**Figure 3:**
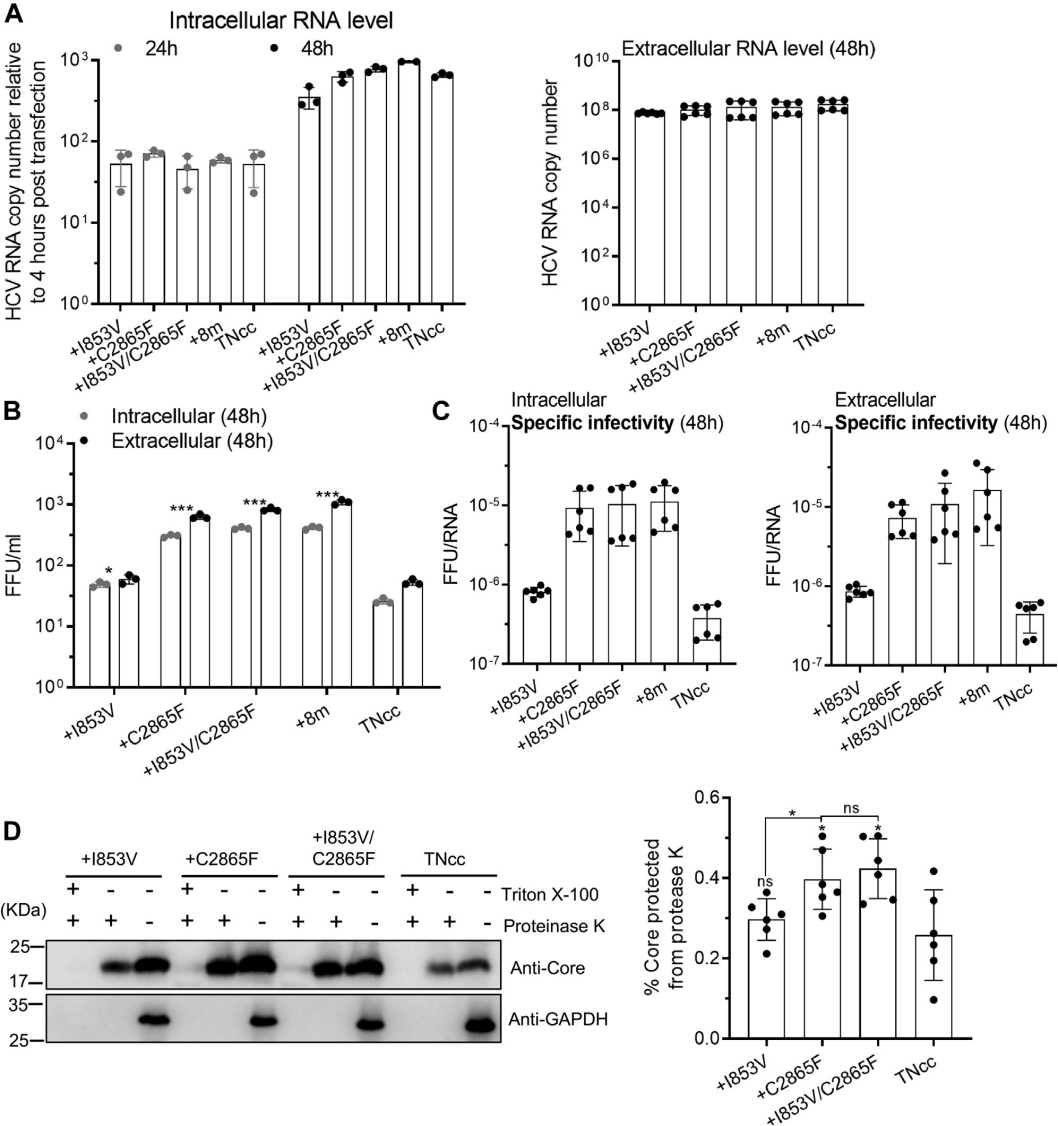
**The combination of I853V and C2865F enhanced the envelopment of viral particle.***A*, equal amounts of RNA transcripts (5 μg) of TNcc with I853V, C2865F, and I853V/C2865F were transfected into Huh7.5.1 cells, in comparison with TNcc and TNcc with eight mutations (8m, Table 1) as controls. Intracellular (*left panel*) and extracellular (*right panel*) HCV RNA levels were measured by RT-qPCR at indicated time points, and intracellular RNA levels were normalized to the RNA levels at 4 h post transfection (input RNA level). *B*, the infectivity titers (extracellular and intracellular) were determined at 48 h post transfection. *C*, the intracellular and extracellular specific infectivity (FFU/RNA) at 48 h. *D*, proteinase K digestion protection assay. Cells were lysed by five cycles of freeze–thaw. The cell lysates were obtained after centrifugation and divided into three equal portions: untreated, treated with 50 μg/ml of proteinase K on ice for 1 h, and pretreated with 5% (vol/vol) Triton X-100 before proteinase K treatment. Digested cell lysates were then analyzed by Western blotting to determine Core protein levels (*D*, *left panel*). The group of proteinase K digestion only (the middle lane of each virus group) was loaded using twice the volume of the nontreatment group (the right lane of each virus group) for a better detection of Core protein, since the Core in the group of proteinase K only was hardly visible in Western blotting if equal volume was loaded in our pilot studies. Statistical analysis of the percentage of Core protected from the proteinase K digestion (*D, right*). Core signals were quantified by ImageJ, and the percentages of protected Core were calculated as the ratio of core in the digested group and the nontreatment control. In *A–C*, data are from three independent experiments and are shown as scatter plots ±SD, and the *D*-right panel was from six independent experiments. Statistics analysis was performed related to the original TNcc (∗, *p* < 0.05; ∗∗, *p* < 0.01; ∗∗∗, *p* < 0.001), unpaired *t* test.

Although I853V and C2865F mutations adapted TNcc to Huh7 cells, those mutant viruses were still more efficient in Huh7.5.1 cells than in Huh7 cells (Fig. 2 and Supporting information Fig. S3). We selected Huh7.5.1 cells to identify the function of I853V and C2865F in the HCV life cycle for the sake of determining RNA levels at early time points after transfection. Equal amounts of RNA transcripts (5 μg) from the original TNcc and TNcc mutants carrying I853V, C2865F, I853V/C2865F, 8m mutations were transfected into Huh7.5.1 cells. Infectivity titers and viral RNA levels were examined. Intracellular viral RNA (24 and 48 h) and extracellular RNA (48 h) post transfection were detected, and intracellular RNA levels were normalized to those at 4 h post transfection (input RNA levels). The results showed that the original TNcc and four mutant viruses were comparable in either intracellular or extracellular RNA levels (Fig. 3*A*). However, the extracellular and intracellular infectivity titers (FFU/ml) of the I853V/C2865F virus were similar to those of the C2865F and 8m viruses but higher than the original TNcc and I853V viruses by ∼10-fold (Fig. 3*B*). Similarly, the specific infectivity of I853V/C2865F, 8m, and C2865F viruses were higher than those of the I853V and TNcc viruses by ∼10-fold (Fig. 3*C*). These results suggest that C2865F and I853V/C2865F mutations did not apparently affect HCV RNA replication but enhanced virus assembly.

Infectious HCV particles contain viral RNA and Core proteins, surrounded by an outer lipid envelope imbedded with E1, E2, and lipoproteins (5, 54, 55). Core proteins will be protected from degradation if virus particles are well enveloped by lipid contents (56, 57). To assess whether the mutations improve the envelopment of HCV assembly, we performed a proteinase K protection assay (58). The results showed that C2865F and I853V/C2865F viruses had more Core proteins protected than TNcc and I853V viruses after proteinase K treatment (Fig. 3*D*). Thus, both C2865F and I853V/C2865F viruses were enveloped in a better form than TNcc and I853V viruses. In conclusion, these data demonstrate that C2865F and I853V/C2865F mutations increased virus production through enhancing virus envelopment.

### C2865F and I853V/C2865F promoted Core translocation from LDs to the ER for efficient viral envelopment

**Figure 4:**
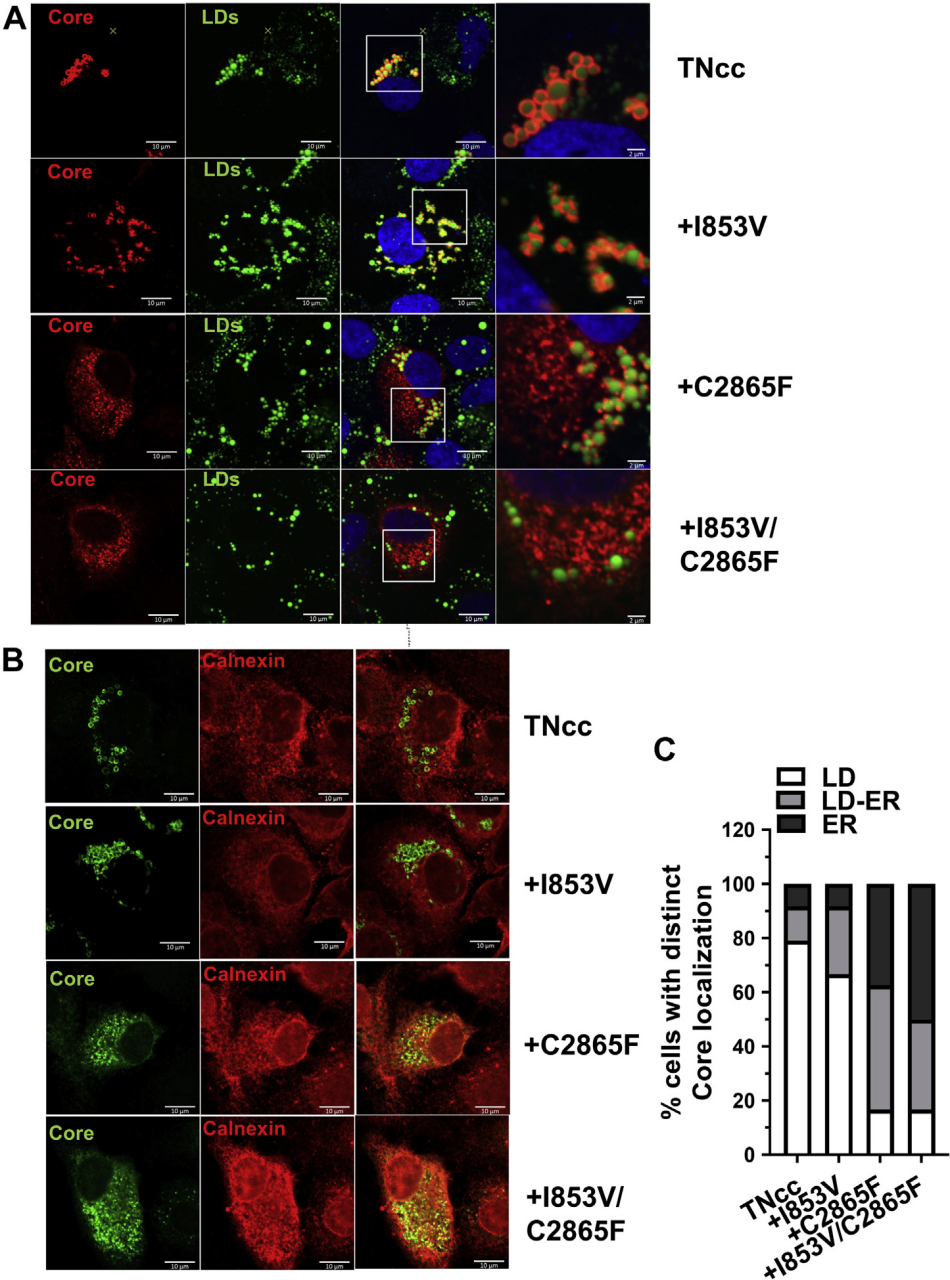
**C2865F and I853V/C2865F facilitated Core translocation from the LDs to the ER.** Equal amounts of RNA transcripts (5 μg) of original TNcc and mutants I853V, C2865F, and I853V/C2865F were transfected into Huh7.5.1 cells. At day 4 post transfection, cells were fixed and subjected to BODIPY 393/503 staining ofLDs (*green*) and immunofluorescence staining of Core (*red*) (*A*) and immunofluorescence staining of Core (*green*) and calnexin for ER (*red*) (*B*). *C*, analysis of Core localization was performed according to the method described by a previous report (35). LD localization was scored when Core proteins were found only at the LD surface without any residual reticular pattern, whereas ER localization was scored when Core staining was found as a reticular pattern with less than 10 LDs fully covered by Core. LD-ER localization was scored when Core was present as a reticular pattern and with more than 10 LDs fully covered by Core. For each virus, ≥20 HCV-positive cells were analyzed and images are representative of three independent experiments.

It is known that Core proteins oligomerize and encapsidate nascent viral RNA on lipid droplets (LDs) and then translocate to the ER assembly site for budding; envelopment occurred when the virus buds from the ER (14). Several studies have shown that the subcellular localization of Core on the ER is crucial for infectious particle assembly (11, 19, 35, 59). Here, we speculated that increased virus envelopment by I853V/C2865F may contribute to Core translocation from the LDs to the ER. We analyzed the LD- and ER-localization of Core proteins for TNcc and mutants I853V, C2865F, and I853V/C2865F by using cytochemistry and immunostaining coupled with confocal microscopy (Fig. 4, *A* and *B*). The percentage of the cells with different patterns of Core subcellular localization (exclusively on either LD or the ER, or found on both organelles) was summarized in Figure 4*C*. The results showed that Core proteins of TNcc and I853V mutants were largely coating LDs (∼75% on LD, ∼15% on ER, and ∼10% on both LD and ER). In contrast, Core proteins of C2865F and I853V/C2865F virus were primarily located on ER or on both LD and ER (∼45% on ER, ∼15% on LD, and ∼40% on both LD and ER). These results suggest that C2865F and I853V/C2865F promoted the subcellular localization of Core proteins from the LDs to the ER, thus facilitating the envelopment of HCV assembly.

### I853V and C2865F mutations enhanced the interaction between NS2 and NS5B

**Figure 5:**
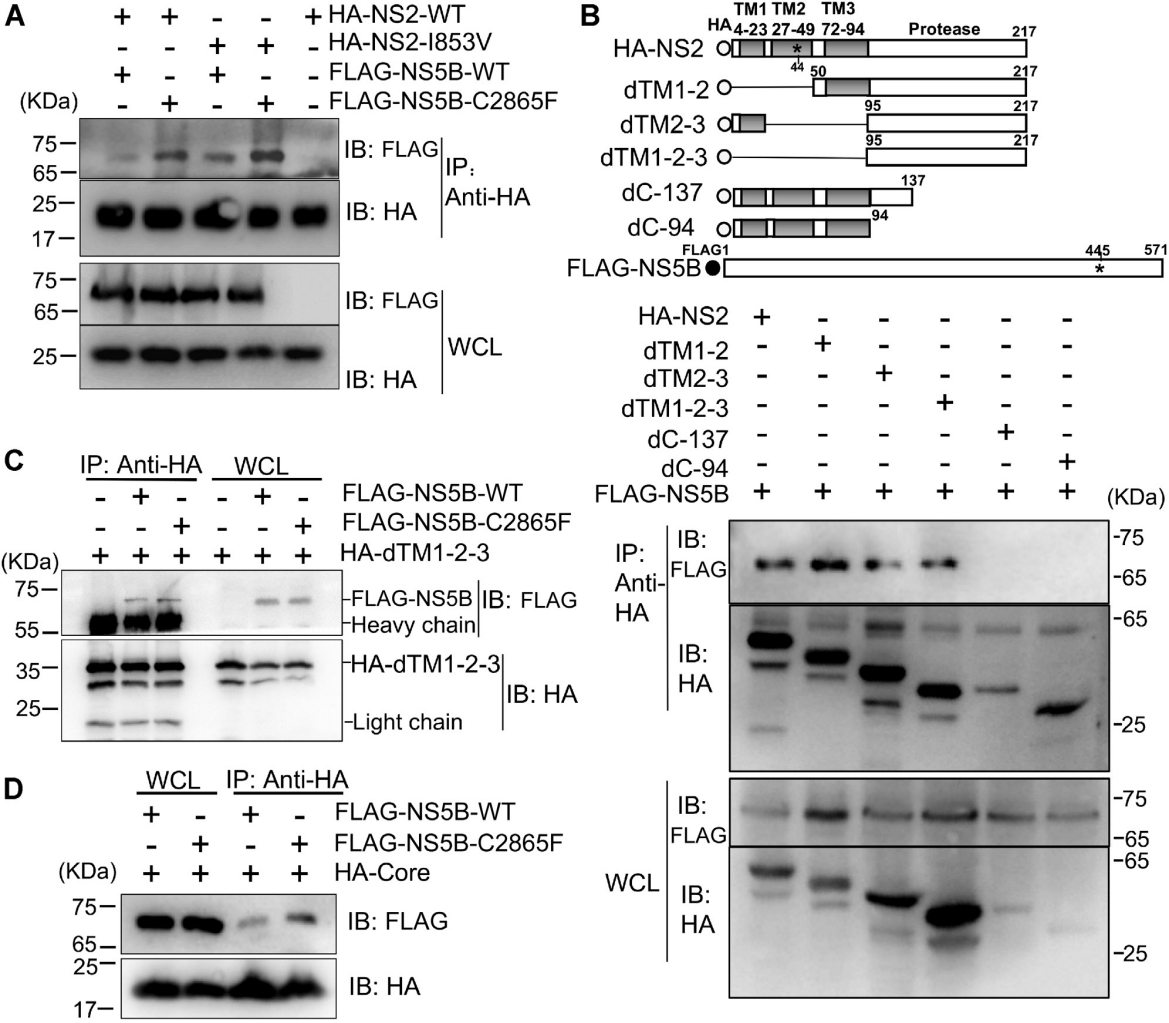
**I853V/C2865F enhanced NS5B interaction with NS2 and Core.** Interactions between NS5B and NS2 with or without mutations were examined by co-immunoprecipitation (Co-IP). *A*, HA-tagged NS2 wildtype (WT) or NS2 with I853V and FLAG-tagged NS5B WT or NS5B with C2865F were cotransfected into 293T cells. Forty-eight hours post transfection, cell lysates of transfected cells were immunoprecipitated with anti-HA antibody. The resulting precipitates and whole cell lysates (WCL) used in immunoprecipitation (IP) were examined by immunoblotting using anti-FLAG or anti-HA antibodies. *B*, interaction of NS5B with truncated NS2 proteins as indicated (dTM1-2, amino acids 50–217; dTM2-3 aa 1–23 and 96–217; dTM1-2-3 aa 95–217; dC137 aa 1–137; dC94 aa 1–94), showing that the NS2 protease domain was responsible for the interaction with NS5B. *C*, FLAG-tagged NS5B WT or NS5B mutant C2865F were cotransfected with HA-tagged dTM1-2-3 into 293T cells, and IP and immunoblotting was performed. *D*, FLAG-tagged NS5B WT or NS5B mutant C2865F were cotransfected with HA-tagged Core into 293T cells, and IP and immunoblotting were performed. Data represent three independent experiments.

The fact that C2865F and I853V/C2865F increased HCV envelopment and Core translocation provided us with clues that the interaction between NS2 and NS5B may be reinforced by mutations. To test this hypothesis, we expressed HA-tagged NS2 wildtype (WT) and mutant NS2-I853V as well as FLAG-tagged NS5B-WT and NS5B-C2865F in 293T cells, and then Co-IP was performed (Fig. 5*A*). The results showed that NS5B interacted with NS2, consistent with a previous report (29). Furthermore, I853V and C2865F individually enhanced the interaction of NS2 and NS5B, but C2865F had a stronger enhancement (Fig. 5*A*).

NS2 plays essential roles in HCV assembly, and TMs have been demonstrated to be involved in HCV assembly by interacting with p7 (11, 19). I853V is located in TM2 of NS2 (corresponding to NS2 aa 44). To investigate which part of NS2 was responsible for the interaction with NS5B and whether I853V and C2865F were directly involved in the interaction, we constructed NS2 with deletions in TMs (dTM1-2, dTM2-3, dTM1-2-3) and protease domain (dC-137 and dC-94) for Co-IP (Fig. 5*B*). We tried to express NS5B with deletions of finger, palm, and thumb domains, but the expression level was marginal; thus, NS5B truncations were precluded in this experiment. The Co-IP experiments revealed that NS5B interacted with dTM1-2, dTM2-3, and dTM1-2-3 but not dC-137 and dC-94. Thus, NS5B interacted with the region of amino acids 138 to 217 in NS2 protease domain. As I853V is in the TM2 domain, not in the protease domain, its enhancement to the interaction of NS2 and NS5B might be achieved by affecting structure of protease domain or by other unknown mechanisms, rather than a direct involvement in the interaction of NS2 and NS5B. Co-IP experiments also showed that C2865F did not enhance the interaction of dTM1-2-3 with NS5B (Fig. 5*C*); thus, C2865F most likely reinforced the interaction of NS2 and NS5B by an indirect mode (Fig. 5*A*). Together, these data suggest that the mutations in NS2 and NS5B did not directly interact with each other but they cooperatively enhanced the interaction between NS2 and NS5B proteins.

Since C2865F accelerated core translocation from the LDs to the ER (Fig. 4), we also examined whether C2865F facilitated the interaction of NS5B and Core. The results showed that NS5B interacted with Core, in agreement with a previous report (60), and C2865F enhanced the interaction of Core and NS5B (Fig. 5*D*). In conclusion, these data demonstrate that adaptive mutations reinforced the interactions of NS5B and Core as well as the NS5B and NS2 protease domain.

### Phenylalanine at position 2865 (2865F) was important for the infectivity of genotype 2a HCV

**Figure 6:**
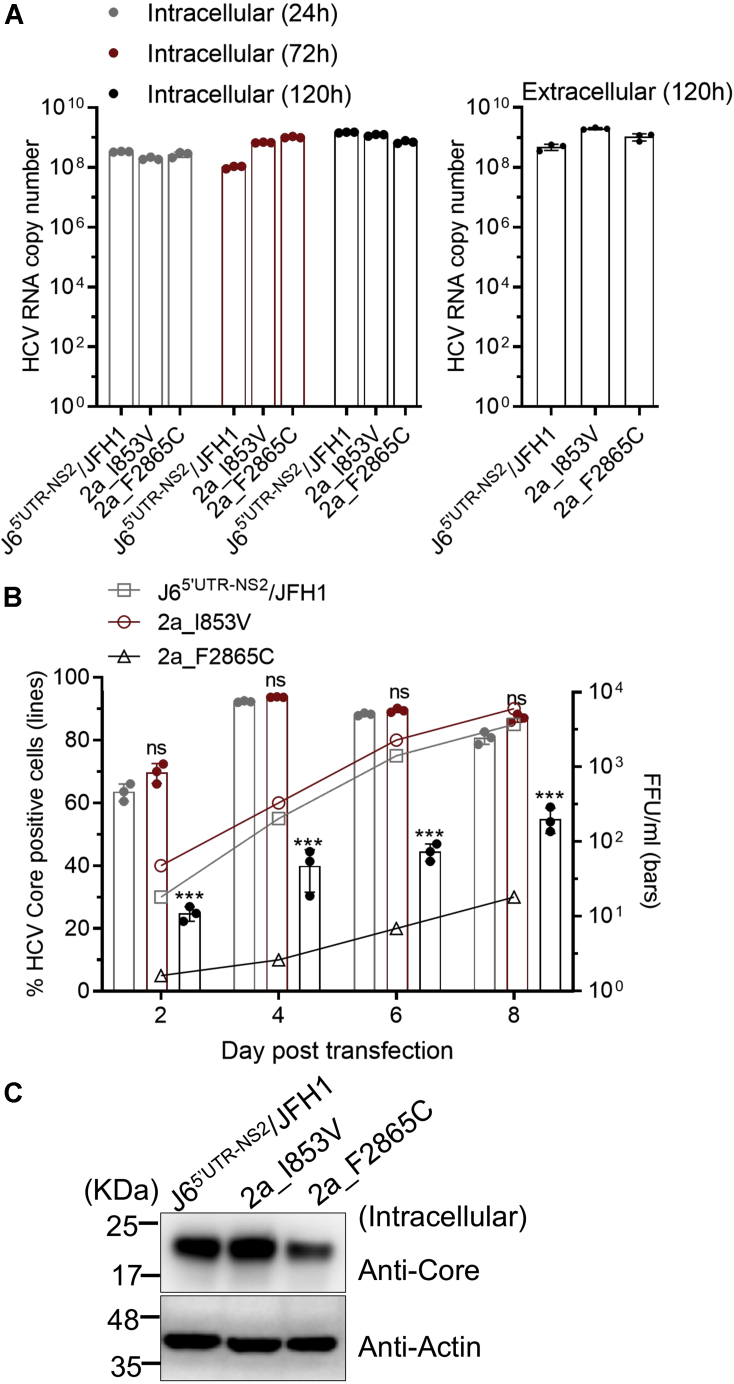
**C2865F increased virus production of a genotype 2a virus.** Five micrograms of RNA transcripts of J6^5’UTR-NS2^/JFH1 (2a), with mutation I853V (2a_I853V) or F2865C (2a_F2865C), was transfected into Huh7.5.1 cells. *A*, intracellular HCV RNA was determined by RT-qPCR at 24, 72, and 120 h post transfection; extracellular viral RNA was determined at 120 h. *B*, percentage of Core-positive cells and infectivity titers. *C*, the intracellular Core proteins were determined at 48 h post transfection by Western blotting. Data are from three independent experiments and are shown as scatter ± SD (*A, B*).

To investigate whether amino acids 853V and 2865F are important for other genotype viruses, we performed a reverse genetics study based on genotype 2a recombinant J6^5′UTR-NS2^/JFH1 (53). We constructed J6^5′UTR-NS2^/JFH1 with I853V and designated it as 2a_I853V. Since 2865F is conserved in full-length genotype 2a clones JFH1 (8), J6cc (9), JFH2 (61), and PR63cc (62), as well as genotype 2b clones J8cc-HT, DH8cc, and DH10cc (9, 47), we mutated the phenylalanine at position 2865 (2865F) of J6^5′UTR-NS2^/JFH1 to 2865C and designated it as 2a_F2865C. After transfection of Huh7.5.1 cells, the original J6^5′UTR-NS2^/JFH1, 2a_I853V, and 2a_F2865C viruses showed little difference in the intracellular HCV RNA levels at 24, 72, and 120 h post transfection as well as in the extracellular RNA levels at 120 h (Fig. 6*A*). Virus spread capacity and infectivity titers of 2a_F2865C were delayed, whereas 2a_I853V was not affected (Fig. 6*B*). 2a_F2865C produced fewer HCV Core proteins at 48 h post transfection (Fig. 6*C*). These data suggest that the enhancement effect of 2865F in HCV production was cross-genotype.

### I853V/C2865F mutant virus showed a density associated with high specific infectivity

**Figure 7:**
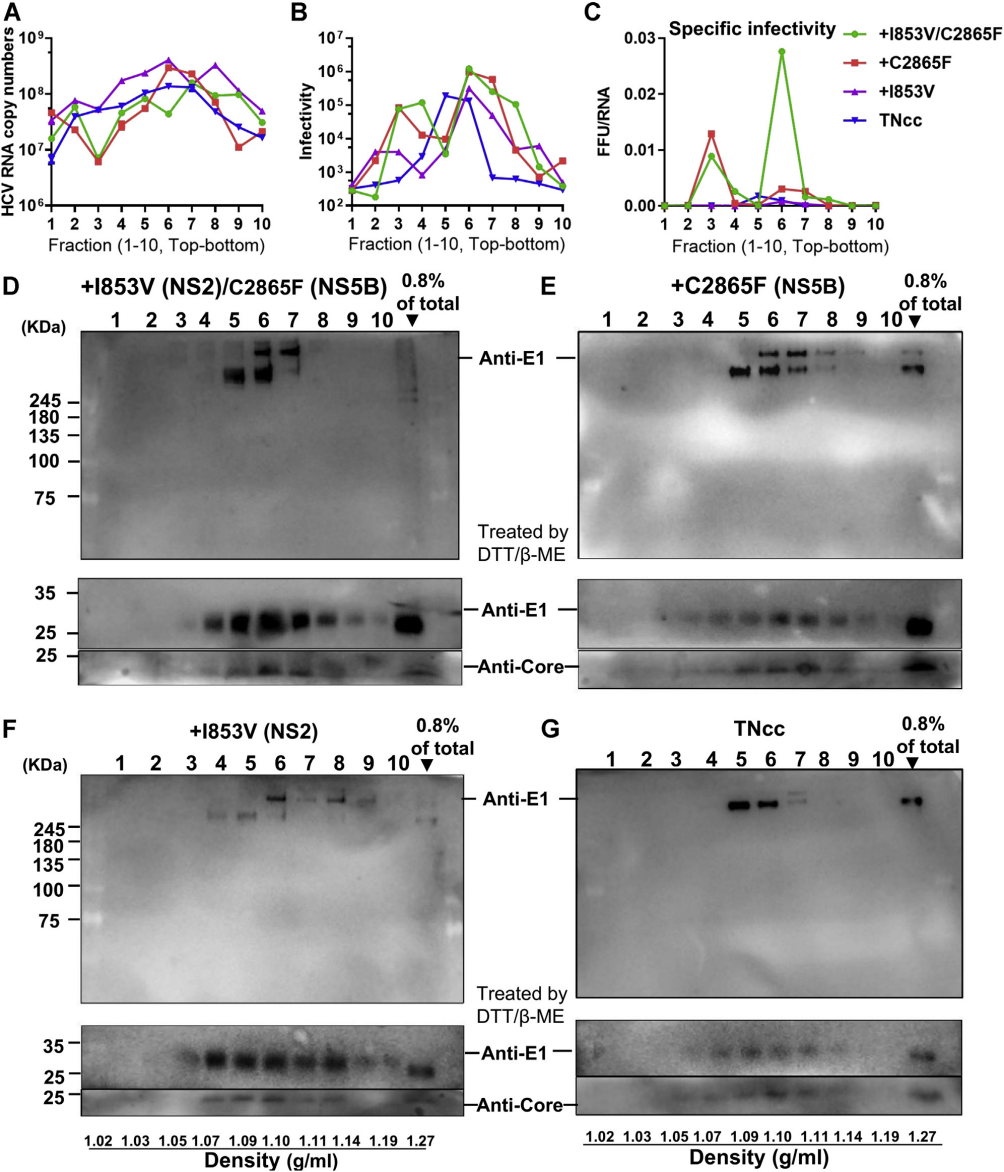
**C2865F and I853V/C2865F promoted the release of HCV particles at a density with high specific infectivity.** Supernatants harvested from Huh7.5.1 cells 4 days post transfection with RNA transcripts of TNcc and TNcc with I853V, C2865F, or I853V/C2865F were concentrated by PEG-8000, fractionated through a 15%–55% iodixanol gradient, and collected into 10 fractions (no. 1–10, from top to bottom of the gradient). *A*, fractions were analyzed by RT-qPCR for HCV RNA copy number. *B*, fifty microliters of each fraction was used to infect Huh7.5.1 cells in a 24-well plate to determine the infectivity titer of fractionated viruses. At 48 h post infection, total RNA was extracted from the cells and intracellular HCV RNA levels were quantified by RT-qPCR. The infectivity titer was defined as the intracellular HCV RNA level according to a previous publication (80). *C*, specific infectivity is defined by infectivity titer/HCV RNA copy number (FFU/RNA copy, mean, n = 3). *D–E*, fractions were analyzed by SDS-PAGE and blotted with anti-E1 antibody in Western blotting (*upper panels*), and fractions were treated with 2-mercaptoethanol and dithiothreitol (DTT) (*middle and bottom panels*) and blotted with anti-E1 and anti-Core antibodies. Concentrated total viral supernatant (0.8%) without fractionation was used as a control (right lane). Data are from three independent experiments.

HCV released to the culture supernatant exists as a pool of infectious and noninfectious particles, of which those with a density of ∼1.10 g/ml have a higher specific infectivity and are more infectious, both *in vivo* (17) and *in vitro* (7, 15, 63). We demonstrated that the I853V/C2865F virus was ∼40-fold higher than the original TNcc in the specific infectivity at an early time point post transfection (48 h) (Fig. 3*C*). Here, we attempted to investigate the buoyant density difference of I853V, C2865F, I853V/C2865F, and the original TNcc viruses (Fig. 7). The culture supernatant was concentrated and separated over an iodixanol gradient. Ten equal fractions were collected, and the viral RNA and infectivity titers were determined for each virus fraction (Fig. 7, *A* and *B*). The specific infectivity of I853V/C2865F virus at fraction 6 (a density of 1.10 g/ml) was much higher than that of other viruses and other fractions (Fig. 7*C*). These data suggest that I853V/C2865F promoted more infectious virion production.

Next, we separated the fractions by SDS-PAGE and blotted them with anti-E1 antibody. The results showed that these viruses had different patterns of the E1 complex (Fig. 7, *D*–*G*). I853V/C2865F and C2865F viruses showed more E1 complex and were concentrated on fraction 6, showing two bands of E1 (different molecular weights) in fractions 5 to 7 and 5 to 8, respectively. I853V virus showed E1 complex in fractions 4 to 8, whereas TNcc had mainly lower bands in fractions 5 to 7 (Fig. 7*G*). HCV with a density of 1.10 g/ml (separated around fraction 6 here) was known to show higher infectivity. Together, these results show that I853V/C2865F and C2865F contributed to the assembly and release of infectious viruses.

The E1-containing complexes were dissociated by reducing agents (DTT and thio-mercaptoethanol) and released monomeric E1 proteins (34 kDa) (Fig. 7, *D–G*, middle panel). We also examined Core proteins and found that Core (17 kDa) was detected and distributed similarly to the pattern of monomeric E1 proteins for each virus (Fig. 7, *D–G*, bottom panel), suggesting that E1 complexes were most likely dissociated from infectious virus particles. Together, these data suggest that the mutations I853V (NS2) and C2865F (NS5B) cooperatively increased the assembly and release of high-specific infectivity HCV particles, with a density of ∼1.10 g/ml.

## Discussion

In this study, we demonstrated that adaptive mutations in NS2 (I853V) and NS5B (C2865F) promoted HCV envelopment of assembly and maturation. We found that mutations C2865F and I853V/C2865F strengthened the interaction of NS2 and NS5B. These mutations also promoted Core translocation from lipid droplets to the endoplasmic reticulum, a process crucial for the envelopment of HCV assembly. The mutations were also found to facilitate the release of HCV particles with a buoyant density of ∼1.10 g/ml.

Culture-adapted mutations play a key role in the development of infectious culture systems for HCV and other viruses, and most of adaptive mutations could initiate and enhance viral RNA replication and virus production (6, 9, 27, 28, 30, 35, 36, 46, 47, 48, 49, 63, 64). Previously, culture adaptation of genotype 2a strain HCV JFH1 or JFH1-based recombinants of various HCV genotypes have identified many different adaptive mutations, and a number of genetic interactions have been studied (23, 27, 28, 31, 32, 33, 34, 36, 63, 64, 65, 66). Many mutations affected virus assembly (23, 27, 28, 31, 32, 33, 34, 36, 63, 64, 65, 66), infectivity (32, 36, 63), viral particle stability (65), and polyprotein processing (33), rather than viral RNA replication. Here, by following the clues from adaptive mutations, we showed that I853V and C2865F cooperatively enhanced virus envelopment of assembly.

The eight mutations identified in this study were not reported previously (Table 1), and the enhancement effect of C2865F and I853V/C2865F was found for genotype 1a and 2a viruses (Figs. 3 and 6). Many studies have shown that the functional role of most of adaptive mutations in the HCV life cycle could vary from one to another. Nevertheless, three key mutations LSG (F1464L, A1672S, D2979G) we previously identified were able to initiate and enhance HCV RNA replication and have become the basis of infectious HCV clones of different genotype viruses (9, 37, 67, 68, 69). Thus, the development of highly infectious HCV clones will identify more adaptive mutations, from which a mutation set may be selected for culture adaptation of any HCV isolates.

The process of ore translocation from the LDs to the ER is crucial for assembly of infectious virus particles (11, 14, 35). All HCV nonstructural proteins compose a complex interaction network (14, 29). Interactions between viral proteins important for the HCV life cycle have been found for NS5A and the LD-bound core (68, 69, 70, 71), NS2 (or p7-NS2 complex) and NS3/4A protease (19, 20), p7 and NS5B (28), as well as NS2 and NS5B (9, 27). Here, our data further our understanding that C2865F and I853V/C2865F promoted Core-ER colocalization, enhanced interactions of NS2 and NS5B as well as Core and NS5B, and facilitated HCV envelopment (Figs. 4 and 5). Protein–protein interactions *per se* may be a signal for curving the ER membrane to bud nascent virus particles. Thus, this study implies that NS5B might be involved in LD-bound Core trafficking to assembly site on the ER by interacting with NS2.

Both serum-derived HCV and culture-adapted HCVcc particles with high specific infectivity have buoyant densities of ∼1.10 g/ml (7, 70), which is significantly lower than other enveloped RNA viruses, because of lipoprotein incorporation (16). HCV particles exist as a mixture of infectious and noninfectious particles in ratios ranging from 1:100 to 1:1000 (6, 7, 17). These mutations hardly affected HCV RNA levels between different fractions and viruses (<10-fold differences). However, I853V/C2865F significantly increased the viral specific infectivity at a buoyant density of ∼1.10 g/ml (fraction 6). Consistent with these results, the E1-containing complex of I853V/C2865F virus had the narrowest range (fractions 5–7) and was concentrated on fraction 6 (Fig. 7D).

The final step of HCV morphogenesis involves the host lipoprotein secretory pathway. HCV virions become mature and associated with the host lipoproteins to form lipoviral particles prior to release (15, 16). The lipid contents determine the density and infectious capacity of HCV virions (71). ApoE not only contributed to virus entry to hepatocytes but also post assembly and viral particle maturation (72, 73). NS5A is reported to interact with apoE, which is crucial for the production of infectious particles by affecting the post assembly stage (74, 75, 76). NS5A also interacts with NS5B, and this interaction is required for HCV RNA replication (77, 78). Since NS5B is involved in HCV pathogenesis (9) and C2865F (NS5B) promotes high-infectivity particle production (Fig. 7), NS5B may be involved in the NS5A–apoE complex to promote the HCV post assembly process. However, further investigation is required.

## Materials and methods

### Ethics statement

This study did not involve human subjects and animal experiments. The use of human hepatoma cell lines was approved by the Medical Ethics Committee at the Zhongshan School of Medicine, Sun Yat-sen University, China.

### Cell lines

Huh7.5 cells were provided by Dr Charlie Rice (Apath L.L.C. and Rockefeller University, New York, USA) (45), Huh7 cells and Huh7.5.1 cells were provided by Dr Jin Zhong and Dr Francis V Chisari (The Scripps Research Institute, La Jolla, USA) (6). HEK293T cells were maintained in our laboratory. All cell lines were maintained in Dulbecco’s modified Eagle’s medium (Life Technologies, USA), supplemented with 10% fetal bovine serum, 100 μg/ml of penicillin, and 100μg/ml of streptomycin (HyClone, USA) in 5% CO_2_ at 37 °C.

### Antibodies

Primary antibodies were anti-HCV Core mouse monoclonal antibody (C7-50) (Santa Cruz Biotechnology, USA), anti-HCV E1 mouse monoclonal antibody, anti-HA mouse monoclonal antibody (MBL, Japan), anti-HA monoclonal antibody (Proteintech, China) for IP, anti-FLAG mouse monoclonal antibody (Beijing Ray Technology, China), anti-Calnexin rabbit antibody (Sigma, USA), and horseradish peroxidase (HRP)-anti-β-actin (Proteintech). The secondary antibodies were goat anti-rabbit IgG-HRP (Proteintech) and goat anti-mouse IgG-HRP (Proteintech) or Alexa Fluor 488 Goat Anti-Mouse IgG (H + L) (Life Technologies). ECL anti-mouse IgG and an HRP-linked whole antibody (GE Healthcare, UK) were used for the FFU assay.

### Plasmids

All plasmids were constructed by standard molecular biology methods, and the constructs were confirmed by DNA sequencing. The TNcc mutants were constructed by overlapping PCR using pTNcc (HCVcc cDNA clone) as a backbone (Table 1) (9). For all Co-IP experiments, expression plasmids were constructed in the background of pcDNA3.1. pTNcc or pTNcc mutants as a template to amplify NS2, NS3, NS5B fragments with HA- or FLAG- tags fused to the N terminus of viral proteins. For better detection, the eGFP protein was fused to the N terminus of the NS2 truncations (dTM1-2, amino acids 50–217; dTM2-3, aa 1–23 and 96–217; dTM1-2-3, aa 95–217; dC137, aa 1–137; dC94, aa 1–94).

### Focus forming unit assay

The infectivity titers of supernatants were determined by FFU assay as previously described (9). Briefly, 24 h before virus infection, Huh7.5 or Huh7.5.1 cells were seeded in 96-well plates at a density of 6 ×10^3^ cells per well. The HCV-containing culture supernatant was diluted, and 100 μl was added to the well and incubated for 48 h. Then, the cells were fixed with cold methanol (−20 °C), immunostained with anti-HCV Core mouse antibody (C7-50) in 1:400 dilutions, and visualized with secondary antibody Alexa Fluor 488 goat anti-mouse IgG (H + L) in 1:800 dilutions. The number of FFUs was manually counted using a fluorescence microscope (Leica Microsystems). The infectivity titers were determined by FFU.

### Transfection and determination of HCV spread, viral RNA, and infectivity titers

Ten micrograms of HCV plasmids were linearized using *Xba*I. *In vitro* transcription and RNA transfection were performed as described previously (46). The transfected cultures were left for ∼16 h and subcultured every 2 to 3 days. The supernatant was collected, filtered, and stored at −80 °C. The percentage of Core-positive cells was determined by immunostaining with anti-Core antibody (C7-50) and Alexa Fluor 488 fluorescence secondary antibody. HCV RNA level was measured by RT-qPCR as previously described (37). Intracellular and extracellular infectivity titers were determined by FFU assay as previously described (9, 37, 64).

### Immunoprecipitation

Plasmids were transfected into HEK293T cells, and 48 h later cells were scraped and lysed with 1× RIPA lysis buffer (10×, 0.5 M Tris-HCl, pH7.4, 1.5 M NaCl, 2.5% deoxycholic acid, 10% NP-40, 10 mM EDTA) containing protease inhibitor cocktail (Sigma, USA) on ice for 30 min. Cell lysates were centrifuged at 12,000*g* for 5 min at 4 °C. The supernatant was transferred to a new tube, anti-HA antibody (Proteintech, China) was added and incubated at 4 °C for 6 h, and then protein A&G agarose beads (Millipore) were added and incubated for another 6 h. The immune complexes were precipitated with the beads by centrifugation at 800*g* for 30 s and washed four times with ice-cold phosphate buffered saline (PBS). The proteins binding to the beads were boiled in 1× SDS sample loading buffer, and the supernatant was subjected to SDS-PAGE.

### Immunoblotting

Cells transfected with plasmids were washed with PBS, scraped, and directly lysed in SDS loading buffer (final concentrations: 75 mM Tris-HCl pH 6.8; 0.6% SDS, 15% glycerol, 0.001% bromophenol blue; 7.5% ß-mercaptoethanol) for 10 min and then boiled at 95 °C for 10 min. The lysates were separated by SDS-PAGE and immunoblotted as described elsewhere (37).

### Protease K protection assay

This protocol was adapted from a previous report (56, 57). Briefly, a portion of Huh7.5.1 cells transfected with *in vitro* transcribed RNA of TNcc and mutants were collected. The cells were resuspended in ice-cold proteinase K buffer (50 mM Tris-HCl, pH 8.0; 10 mM CaCl_2_; 1 mM DTT). Cells were then lysed by five cycles of freeze–thaw cycles. The cell lysates were obtained after centrifugation and divided into three equal groups: untreated, treated with 50 μg/ml of proteinase K on ice for 1 h, and pretreated with 5% (vol/vol) Triton X-100 before proteinase K treatment. Proteinase K was then inactivated with 5 mM phenylmethylsulfonyl fluoride on ice for 10 min. The samples were mixed with 4× SDS sample buffer (1 M Tris pH 6.8, 60% glycerol, 0.06% bromophenol blue, 12% SDS), incubated at 90 °C for 5 min, and immunoblotted for HCV Core and GAPDH protein.

### Immunostaining and confocal microscopy assay

HCV-infected cells grown on slides were fixed with 4% paraformaldehyde for 20 min at room temperature, treated with 0.2% Triton X-100 in PBS for 10 min, and blocked with 1% bovine serum albumin in PBS for 30 min. Subsequently, the cells were incubated with the primary antibody in 1% bovine serum albumin–PBS overnight, washed, and stained with the secondary antibodies conjugated with Alexa 488 (Life Technologies) or Alexa 555 (Life Technologies) for 2 h. The LD staining was performed using a specific cellular tracer of neutral lipids, BODIPY 493/503 (Life Technologies). The slides were mounted with Prolong Anti-fade (Life Technologies). Images were captured using a confocal microscope (Zeiss, LSM800) and processed using Adobe Photoshop CS6 software. The Core subcellular localizations were quantified as previously described (11, 35). Briefly, 10 to 20 HCV-infected cells were randomly selected for determination. LD localization was scored when Core proteins were found only at the LD surface without any residual reticular pattern, whereas ER localization was scored when Core staining was found as a reticular pattern with less than 10 LDs fully covered by Core. Both LD and ER localization (LD-ER) was scored when Core was present as a reticular pattern and with more than 10 LDs fully covered by Core protein.

### Quantitative detection of HCV RNA

Viral RNAs were isolated from centrifugation-clarified cell supernatants or cell pellets using TRIzol reagent (Magene, China). RNA (500 ng) was used for reverse transcription using HiScript II Q RT Super Mix kit (Vazyme, China) following a thermal cycle of 42 °C for 2 min, 55 °C for 15 min, and 85 °C for 5 s. One microliter of cDNA was subjected to quantitative PCR by using a StepOne qRT-PCR SYBR Green PCR Kit. The HCV-specific primers used in quantification were as follows: HCV Sense: 5′-CTTCACGCAGAAAGCGCCTA- 3′ and HCV anti-Sense: 5′-CAAGCGCCCTATCAGGCAGT-3′. PCR was performed by one cycle of 95 °C for 5 min, followed by 40 cycles of 95 °C for 15 s and 60 °C for 1 min. The amount of HCV RNA was calculated by a standard curve made from a serial dilution of a full-length HCV genomic plasmid, of which the copy number of DNA molecules was quantitated.

### Biochemical subcellular fractionation

Polyethylene glycol (PEG)-concentrated supernatants were separated through a 15% to 55% iodixanol gradient as previously described (58, 79). Briefly, 4 days post transfection of Huh7.5.1 cells with HCV RNAs, the supernatant was collected, mixed with PEG-8000 to a final concentration of 8%, and incubated with gentle rocking at 4 °C overnight. PEG-containing supernatants were centrifuged at 10,000*g* for 30 min, and pellets were collected and resuspended in ice-cold 1× PBS. The resuspension was loaded over a 15% to 55% iodixanol gradient and centrifuged at 172,000*g* for 16 h in a Beckman SW55 Ti rotor. Ten fractions (0.46 ml per fraction) were collected. Fraction one had the lowest density (∼1.02 g/ml), and each fraction was measured for HCV infectivity titers (FFU/ml), HCV RNA copy numbers (RT-qPCR), and E1 and Core proteins (Western blotting). In some experiments (indicated in figure legends), the protein collected from the gradients was incubated in 2× urea loading buffer (50 mM Tris-HCL, 1.6% SDS, 7% glycerol, 8 M Urea, 4% 2-mercaptoethanol, bromophenol blue) at 37 °C with vortex every 10 min for a total of 30 min. It was then boiled at 95 °C for 10 min prior to SDS-PAGE and immunoblotting.

## Data availability

Raw sequencing data and other data used for statistical analysis are available upon request (Dr Yi-Ping Li, Zhongshan School of Medicine, Sun Yat-sen University. Email: lyiping@mail.sysu.edu.cn).

## Conflict of interest

The authors declare that they have no conflicts of interest with the contents of this article.
