# Supplementary material for: Adaptive mutations promote hepatitis C virus assembly by accelerating core translocation to the endoplasmic reticulum
Source: J Biol Chem. 2020 Nov 23;296:100018. doi: 10.1074/jbc.RA120.016010 (PMC7949066; doi:10.1074/jbc.RA120.016010)
Supplement: Supplementary Figs [file mmc1.pdf]

Adaptive mutations promote hepatitis C virus assembly by accelerating Core translocation to the endoplasmic reticulum

Fuxiang Zheng[1, 5], Ni Li[1], Yi Xu[2], Yuanping Zhou[3], Yi-Ping Li[1, 4 *]

Supporting information

Figure S1

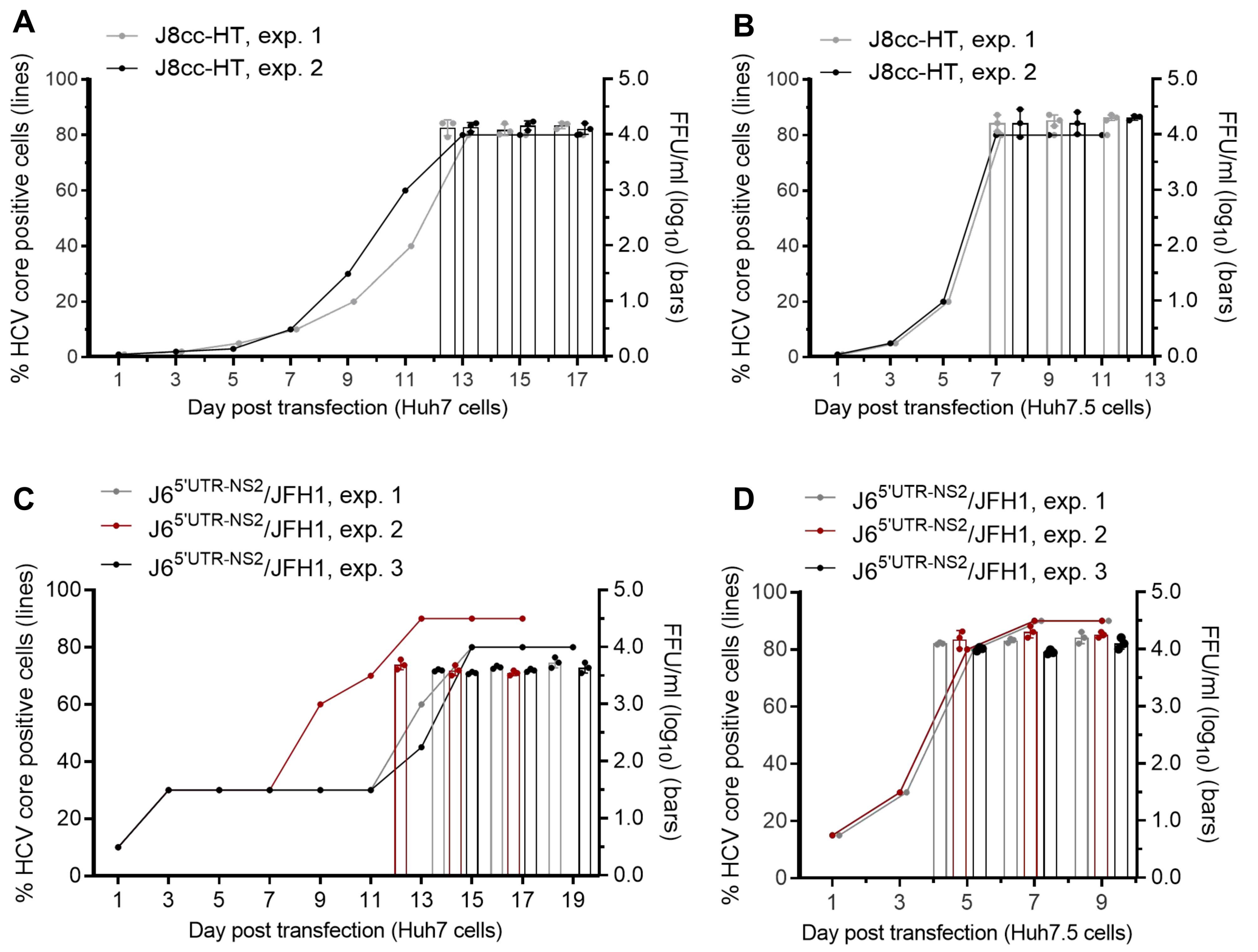

Fig. S1. Virus spreads of genotype 2b (J8cc-HT) and 2a (J6$^{5'UTR-NS2}$/JFH1) were delayed in Huh7 cells. Equal amounts (5 µg) of RNA transcripts of 2a J8cc-HT and 2a J6$^{5'UTR-NS2}$/JFH1 recombinants were transfected into Huh7 cells and Huh7.5 cells. (A) In Huh7 cells, the percentages of Core-positive cells (lines) and infectivity titers (bars) of 2b J8cc-HT virus were monitored at days 13, 15, and 17 post transfection. Virus spread was delayed by approximately one week and infectivity titers were 5-fold lower than those in Hun7.5 cells ($10^{4.5}$ FFU/ml) (in panel B). (C) In 2a J6$^{5'UTR-NS2}$/JFH1-transfected Huh7 cells, delayed spread was also observed; however at peak infection the infectivity titers were comparable to that in Huh7.5 cells (10^4 FFU/ml) (in panel D). Data are from three independent experiments and are shown as scatter ± SD.

Figure S2

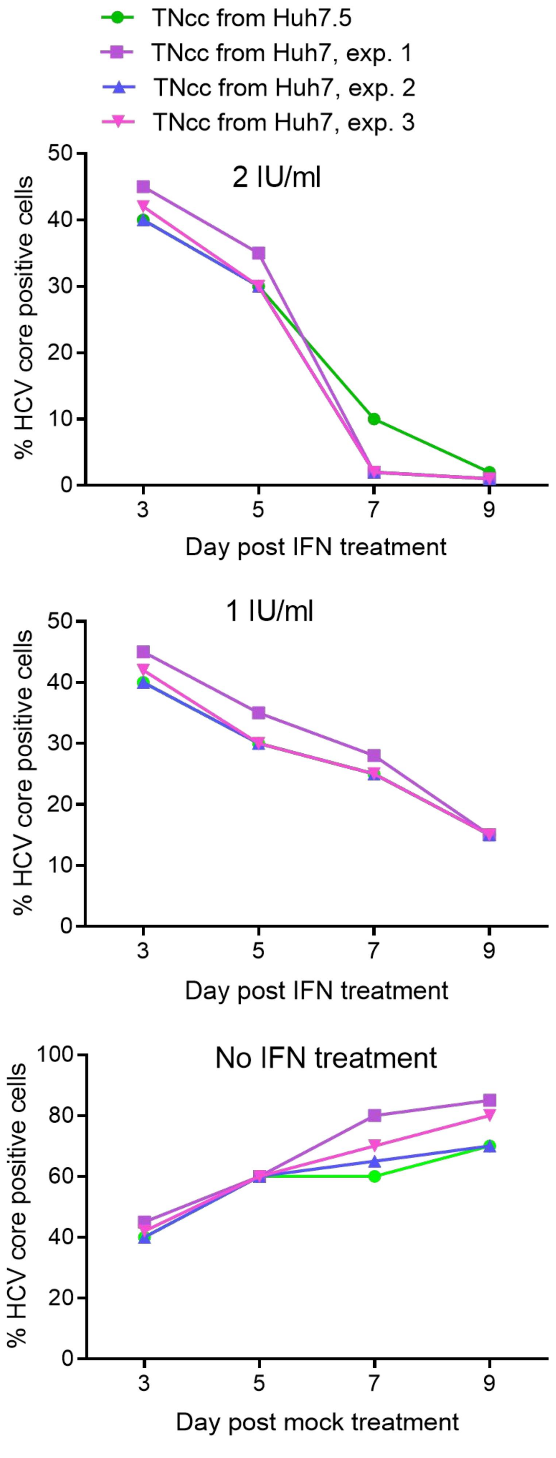

Fig. S2. Huh7-adapted TNcc viruses did not confer resistance to IFN treatment. TNcc virus adapted to Huh7 cells collected from three experiments of long-term culture adaption (Fig. 1A) were tested for viral sensitivity to interferon treatment, in comparison with TNcc propagated from

Huh7.5 cells. Huh7.5 cells were infected with TNcc viruses (MOI=0.5), and interferon-α was added into the medium at the indicated concentrations (2 IU/ml, 1 IU/ml, and no IFN treatment). The percentage of HCV Core-positive cells were determined. Data are from three independent experiments and are shown as the means.

Figure S3

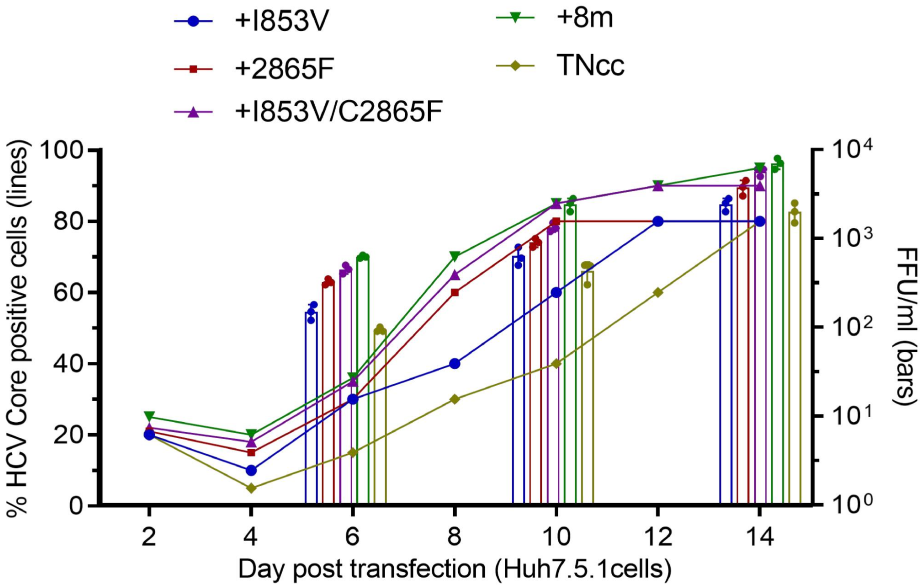

Fig. S3. TNcc and mutant virus spread efficiently in Huh7.5.1 cells. Equal amounts (5 μg) of RNA transcripts of TNcc with I853V, C2865F, and I853V/C2865F were transfected into Huh7.5.1 cells, in comparison with TNcc and TNcc with eight mutations (8m, Table 1). The percentage of Core-positive cells and the infectivity titers of supernatant were determined at the indicated time points. Data are from three independent experiments and are shown as scatter ± SD.
